# Collaborative Localization Algorithms for Wireless Sensor Networks with Reduced Localization Error

**DOI:** 10.3390/s111009989

**Published:** 2011-10-21

**Authors:** Prasan Kumar Sahoo, I-Shyan Hwang

**Affiliations:** 1 Department of Computer Science and Information Engineering, Chang Gung University, Kwei-Shan, 33302, Taiwan; 2 Department of Information Communication, Yuan-Ze University, Chungli, 32003, Taiwan; E-Mail: ishwang@saturn.yzu.edu.tw; 3 Department of Computer Science and Engineering, Yuan-Ze University, Chungli, 32003, Taiwan

**Keywords:** wireless sensor networks, localization, error estimation

## Abstract

Localization is an important research issue in Wireless Sensor Networks (WSNs). Though Global Positioning System (GPS) can be used to locate the position of the sensors, unfortunately it is limited to outdoor applications and is costly and power consuming. In order to find location of sensor nodes without help of GPS, collaboration among nodes is highly essential so that localization can be accomplished efficiently. In this paper, novel localization algorithms are proposed to find out possible location information of the normal nodes in a collaborative manner for an outdoor environment with help of few beacons and anchor nodes. In our localization scheme, at most three beacon nodes should be collaborated to find out the accurate location information of any normal node. Besides, analytical methods are designed to calculate and reduce the localization error using probability distribution function. Performance evaluation of our algorithm shows that there is a tradeoff between deployed number of beacon nodes and localization error, and average localization time of the network can be increased with increase in the number of normal nodes deployed over a region.

## 1. Introduction

In recent years, with rapid advances in Micro Electro Mechanical Systems (MEMS) technology, research on Wireless Sensor Networks (WSNs) has received extensive interest. It is getting popular due to its low cost and small size and its applications in military and civilian surveillance. However, wireless sensor networks have a few inherent limitations. e.g., limited hardware, limited transmission range, and large scale network system and the traditional protocols or mechanisms cannot use in WSNs. Hence, several issues are needed to consider in WSNs to construct an efficient and robust network. For example, sensor nodes have limited computation capability and limited power supply, and therefore low complexity algorithms and power saving schemes should be designed.

In wireless sensor networks, localization of nodes plays an important role in most of the applications. When sensors are deployed over a network, normally they have only connectivity information with their neighbors, without knowing their own location information. In some situations, the problem can have easy solution if location information of the nodes is available. For example, routing path can be constructed easily, and coverage hole can easily be detected, if nodes have location information. Knowing relative location of sensors allows the location-based addressing and routing protocols, which can improve network robustness and energy-efficiency effectively. Recent research results show that nodes with location information lead to increased performance of applications and reduced power consumption. In addition, more accurate location information leads to the more accurate result that application needs. In summary, localization is an essential part of WSNs.

Sensors are intended to be low-cost disposable devices, and currently developed solutions such as global position system (GPS) [1] are inadequate for the hardware and power-limited sensors. Traditional localization techniques are not well-suited for these requirements. Besides, a global positioning system (GPS) receiver on each device is cost and energy prohibitive for many applications, not sufficiently robust to jamming for military applications, and limited to outdoor applications. Local positioning systems (LPS) [2] rely on high-capability base stations being deployed in each coverage area, and is an expensive burden for most low-configuration wireless sensor networks. Hence, automatic localization of the sensors in wireless networks is a key enabling technology. The overwhelming reason is that a sensor’s location must be known for its data to be meaningful. As an additional motivation, sensor location information can be extremely useful for scalable, and geographic routing algorithms.

In wireless sensor networks, localization is an important task that refers to the ability of determining relative or absolute position of sensor nodes with an acceptable accuracy. Collaboration among nodes is highly essential so that localization can be accomplished by the nodes themselves without any human intervention. In WSNs, normally such collaboration occurs among nodes located in a certain region. In this paper, we propose the localization of sensors through collaboration among nodes to minimize the localization error and to find localization accuracy as much as possible. In our localization algorithms, the normal, beacon and anchor nodes collaborate with each other to calculate the location information of the nodes by considering several aspects like limited energy resource, number and density of nodes and existence of obstacles. A novel localization scheme along with localization error determination and correction methods are also proposed to calculate the relative location of the nodes in a collaborative manner with help of anchor and beacon nodes. The main contributions of our work can be summarized as follows.

- We combine the range-free and range-based localization schemes to determine the location of normal nodes distributively by using limited beacon nodes with location information. Due to the use of fewer beacon nodes, our algorithm could be cost effective.
- In most of the localization algorithms, a free space model is considered for the propagation of signal, which is an over idealization case. Since noise and obstacle must affect the localization system, we develop localization algorithms that consider the fading and shadowing effect. Hence, our localization model can be useful for both outdoor and indoor environment.
- In range-based localization schemes, a node has to depend on the location information of other nodes to determine its own location and all most all localization schemes are probabilistic by nature. Hence, error must be incurred as a node may receive location information from more than one beacon nodes. In this work, analytical methods are developed using probability distribution function to find out the probability of wrongly identifying a transmission arriving from a node with location information.
- Analytical methods are developed to reduce the localization error and therefore our localization system can provide more accurate location information of a node.

The rest of the paper is organized as follows. An overview of the related work is presented in Section 2. Our proposed localization algorithm, localization error determination and correction methods are described in Section 3. Performance evaluation of our algorithms is presented in Section 4 of the paper and concluding remarks are made in Section 5.

## 2. Related Work

Localization in wireless sensor networks is different from the traditional wireless communication technology. It is an important aspect in WSNs as the events detected by sensors usually should contain location of those nodes that detect a target. For example, location of a military tank should be informed to the sink if it is detected by the sensors, which can be achieved through location information of the sensors. Besides, many network operations also depend on the locations of sensors, such as geographic routing, key distribution protocols, and location-based authentication. Incorrect locations may lead to severe consequences. For example, lack of location information of sensors may lead to wrong military decisions on the battlefield and falsely granting access rights to people. Thus it is important and essential to ensure the correctness of sensors’ locations. There has been an increasing interest in the localization techniques for WSNs in recent years and many localization algorithms [3–5] have been proposed. Constraint on limited hardware supports and power supply, sensor nodes can only find its approximate location information. In order to find node’s location effectively, various localization algorithms are proposed, which can be further classified into *range-based* and *range-free* localization schemes. The range-based localization scheme [6] uses measurements of distance or angle to estimate the node’s location. According to signal propagation and receive time, two kinds of technology are mentioned to obtain the distance. They are: time of arrival (TOA) [7], time of difference of arrival (TODA) [8]. TOA method is used to obtain the range between the sender and receiver nodes by signal arrival time. TODA technique is based on the difference in time between two different signals arrival time and is widely proposed as a necessary measurement method in localization solution for WSNs.

The algorithms proposed in [9] and [10] are self-organized methods to establish the relative coordinate system on every known nodes through the TODA. Angle of arrival (AOA) technique [11] is another ranged-based localization algorithm. In this algorithm, normal nodes have ability to detect the angle to neighbor nodes by directional antenna or smart antenna. Using the angle information, location of the nodes can also be calculated. However, these three methods require additional equipments and hardware supports, which may incur additional cost and energy consumption. Hence, these protocols seem less suitable for the low-power WSNs. With help of global position system (GPS), few beacon nodes can obtain their absolute location information and other unknown nodes estimate their location information by receiving the beacon packets from the beacon nodes. In [12], authors propose a localization scheme called approximate point in triangular test (APIT) algorithm. In APIT, each beacon node first broadcasts the beacon packet to the neighbor nodes, which is later flooded into the whole network. Then each unknown node determines if it is within a particular triangle formed by a set of beacon nodes. Finally, an unknown node estimates its location by the center of gravity of the overlapped area. Although location information of the unknown nodes can be obtained by this algorithm, still some problems exist in it. First, the accuracy relies heavily on the percentage of beacon nodes, and communication cost is high as each node needs to listen many times to different beacon packets. Besides, the complexity of computations is high when the unknown node estimates the overlapped area.

A range-free localization scheme called distance vector hop (DV hop) is proposed in [13,14]. It uses topological information and number of hops alternative to the real distance. In the beginning, the beacon node floods the packet with hop count and node ID to the rest of the network. Unknown nodes compute the average hop size of their nearest beacon nodes, translate the number of hops into real distance and estimate their position. However, some drawbacks exist in DV-hop algorithm, since localization accuracy depends on the node density. Besides, irregular deployment will cause the inaccuracy of average hop size and communication cost is still high. In order to improve the accuracy of location information, a distributed location estimation scheme (DLS) is proposed in [15]. In this algorithm, each beacon node exchanges the node ID and location information to all nodes of the network. The unknown node calculates its own estimated rectangle (ER) and regards the center of ER as its location. In [5], the authors propose a distributed range-free algorithm, called Concentric Anchor Beacon (CAB) localization algorithm. In CAB, each beacon node emits several beacon packets with different power levels and each node maintains a table that includes the ID, location, transmit power level and constraint region of the beacon node. Each normal node determines the particular ring or circle it belongs to within range of different anchors. From the intersection points of different rings, the average of those intersection points is estimated as the location of a node.

Although CAB uses few beacon nodes for localization, it has still some drawbacks. Firstly, it is not a good method for beacon nodes to transmit packets with different power level. Moreover, averaging the intersection point is not accurate result. If some nodes have same intersection points, then the algorithm will give same location information to those nodes. From the discussion of those two kinds of localization schemes, it is clear that each of them have unique properties. In range-based scheme, it can provide more accurate location estimation, but needs additional equipments. In range-free scheme, low cost location system can be built, but estimated location is not accurate enough than range-based schemes. The lognormal shadowing model is used [16] in wireless sensor networks to analyze the path loss characteristics versus distance relationship through a distance power exponent and random shadowing effects. Subsequently the model is used in their work to synthesize the propagation environments.

The authors in [17] propose a localization estimation scheme for the wireless sensor networks in Non-Line-of-Sight environments, where appropriate signal strength is not received due to multi-path fading and shadowing. Though they have considered the lognormal shadowing model in their work, they have used four beacon nodes to estimate the location of a normal node without taking the possible localization error into account. The relationship between the Received Signal Strength Indication (RSSI) values and distance in wireless sensor networks is analyzed in [18] taking lognormal model. They use the lognormal shadowing model in wireless sensor networks to estimate the coefficients in the model, which could be dynamically adjusted with the changed environments. In our work, we design algorithms to find location of normal nodes with help of the location of few beacon nodes. We consider the lognormal shadowing model in our protocol as it affects the received signal strength. The detail procedures of our localization methods are described in the following sections.

## 3. DIstributed Localization (DIL) Algorithm

**Figure 1.**
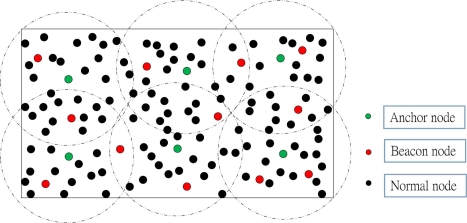
Example of node deployment strategy in the localization system.

In this section, we propose our DIstributed Localization (DIL) algorithm, where a rectangular outdoor monitoring region is considered to find the location of the nodes. In our localization scheme, all deployed nodes are classified as *Normal*, *Beacon* or *Anchor* nodes to locate the position of the normal nodes. Normal nodes and beacon nodes are deployed randomly over the monitoring region, where normal nodes have no location information. However, beacon nodes have location information with higher capability of computation and energy resource. Anchor nodes have larger communication range and are deployed manually. In our protocol, it is assumed that anchor nodes provide angle information to each normal nodes of the network and percentage of anchor nodes is less than the beacon nodes. As shown in Figure 1, the whole network is divided into several clusters such that only one anchor node can be available in each cluster. In order to ensure that each normal node gets enough information to calculate its location, there must be at least one beacon node in each cluster. Otherwise, additional beacon nodes should be redeployed in each cluster. Though more than one beacon node may be available in each cluster, incoming packets from at most three different beacon nodes are used to find the location of a normal node. The deployment strategy of those three types (anchor, beacon and normal) of nodes is described in the following subsection.

### 3.1. Node Deployment Strategy

In our localization system, normal nodes should receive enough information from the beacon and anchor nodes to calculate their location information correctly. In order to ensure every normal node get enough information, it is necessary to design a proper node deployment strategy so that nodes can be localized efficiently. As per our assumption, normal nodes get angle information from the anchor nodes. Hence, first the anchor nodes are deployed randomly to make sure that the entire monitoring region is fully covered. It is assumed that the size of the monitoring region is *m* × *n*, and communication range of the anchor node is *R_c_* = 2*R_s_*, where *R_s_* is the sensing range of each normal node. It is to be noted that each anchor node knows its location via GPS.

A small percentage of beacon nodes that is more than the number of the anchor nodes are deployed on the monitoring region randomly after deployment of the anchor nodes. After random deployment of the anchor and beacon nodes, higher percentage of the normal nodes that is more than the number of the beacon nodes are deployed randomly. Hence, in our localization scheme, it is assumed that percentage of *Anchor* nodes *<* percentage of *Beacon* nodes *<* percentage of *Normal* nodes.

### 3.2. Localization Algorithm

In the localization algorithms, first the distance measurement mechanism of the normal nodes is introduced from the received signal strength indicator (RSSI) value of the beacon nodes. The algorithms are proposed to compute the coordinate of each normal node based on the angle information from an anchor node and distance information from at most three beacon nodes taking the fading and shadowing effects due to noise and obstacles, as described below.

#### Distance Measurement

The received signal strength indicator (RSSI) is one type of distance estimation technology to obtain the distance between the transmitter and receiver [19,20]. This measurement technology is a standard feature found in most wireless devices and is attractive as they do not need any additional hardware support. When the transmitter sends packet to the receiver, receiver gets the RSSI value as the inverse square of the distance. In most of the localization algorithms, the propagation of signal is considered as an over idealization case, e.g., free space model. However, because of the noise and obstacle, the fading and shadowing effects must be considered in node localization. Experimental results [21] show that many well-designed protocols in WSNs fail in a realistic wireless environment. Typically, the mean RSSI decays between the transmitter and receiver (T-R) can be predicted by some radio propagation model. Because of multi-path fading and shadowing, the received signal strength in wireless channel cannot be obtained appropriately and location estimation error is inevitable. However, log-normal shadowing model can better describe the relationship between the RSSI value and distance. Hence, the log normal shadowing model is a most commonly used propagation model that considers the shadowing effect, whether in outdoor or indoor environment. This model indicates that decrease in average received signal strength with distance is logarithmical. In general, the average path loss for an arbitrary T-R separation can be expressed as given in Equation (1),

(1) $$P_{r}\left(d\right)=P_{t}\left(d_{0}\right)-10n\log\left(\frac{d}{d_{0}}\right)+X_{\sigma }$$

where *n* is the path loss exponent, which depends on the specific propagation environment, *d* is the distance between T-R, *P_r_*(*d*) represents the received signal strength indicator (RSSI), and *P_t_*(*d*_0_) represents the transmission power at reference distance (*d*_0_). The term *X_σ_* is a random variable, which accounts for the random variation of the shadowing effect and is supposed to be Gaussian distribution with zero mean random variable (in dB) with standard deviation *σ* (also in dB). Based on Equation (1), we can obtain the distance *d* from Equation (2).

(2) $$d=d_{0}*10^{-\left(\frac{P_{t}\left(d_{0}\right)-P_{r}\left(d\right)-X\sigma }{10n}\right)}$$

#### Coordinate Computation

After measuring the distance between the beacon and normal nodes based on the RSSI value, coordinate of each normal node can be calculated. According to our assumptions, a normal node must have received location information from at least one beacon node and angle information from only one anchor node to calculate its own location. To satisfy the conditions of the assumption, a normal node must wait for a predefined timeout *T_n_* units to receive RSSI value from at least one and at most three beacon nodes. During the waiting time, if a normal node does not receive any RSSI value from at least one beacon node, it has to again wait for the *T_n_* units and the process continues till a normal node receives RSSI value from any beacon nodes within its communication range. Upon receiving RSSI value from the beacon nodes and angle information from the anchor node, a normal node starts calculating the possible coordinates of its own location.

**Figure 2.**
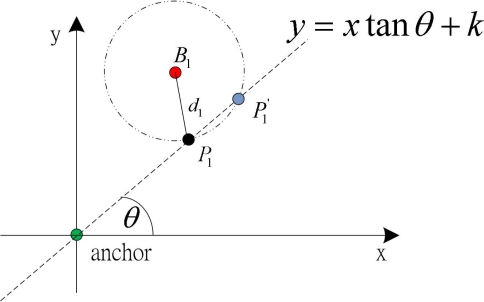
Location computation of a normal node with help of one beacon node.

Let (*x, y*) be the coordinate of the normal node, (*x*_1_, *y*_1_) be the location of a beacon node *B*_1_, and (*x_a_, y_a_*) be the location of an anchor node. Distance between the beacon and normal node is *d*_1_, which is calculated as described later. Let the angle between the anchor node’s *x*-axis and the line joining the normal and anchor node be *θ*. Based on these information, we can obtain two equations. We consider the linear equation that passes through the anchor and normal node, as shown in Figure 2 and given in Equation (3).

(3) y=xtanθ+k

where *k* is a constant, which is obtained by substituting location of the anchor node.

(4) $$k=y_{a}-x_{a}\tan\theta$$

Taking communication range of a beacon node as a uniform circular disc, Equation (5) can be obtained as follows.

(5) $${\left(x-x_{1}\right)}^{2}+{\left(y-y_{1}\right)}^{2}=d_{1}^{2}$$

Substituting Equation (3) in Equation (5) and upon simplification, Equation (6) can be obtained.

(6) $$\left(1+\tan^{2}\theta \right)x^{2}-\left({2x}_{1}+{2y}_{1}\tan\theta -2\mathrm{ktan}\theta \right)x+R=0$$

where, *R* is

(7) $$R=x_{1}^{2}+k^{2}-{2\mathrm{ky}}_{1}+y_{1}^{2}-d_{1}^{2}$$

Since Equation (6) is a simple quadratic equation, *x* coordinate of the location of the normal node can be calculated easily. Now *y* coordinate of the location can be obtained by substituting value of *x* in Equation (3). However, it could be possible that a normal node may receive beacon packets from two or more beacon nodes. It is to be noted that each normal node can have at least one beacon node within its communication range as per our assumptions. As described previously, first a normal node listens to the network and checks the arrival of the beacon packets. Normal node continues to wait for *T_n_* units and maintains a coordinate table as shown in Table 1 to record the beacon packet’s information. Each normal node maintains the coordinate table with four fields. They are the ID, location and RSSI value of the beacon nodes from which beacon packets are received. Besides, the last column of the table records the possible estimated location (P-Loc) information of the normal node. Normally, a normal node computes the possible location (P-Loc) from all of its received data as soon as its waiting time expires.

**Figure 3.**
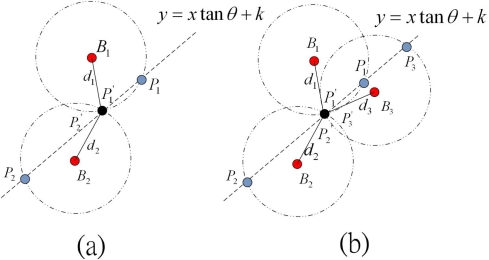
Location determination of normal node: **(a)** with help of two beacon nodes, **(b)** with help of three beacon nodes.

For example, suppose a normal node receives beacon packet from two different beacon nodes *B*_1_ and *B*_2_. As shown in Figure 3(a), from the sensing range of *B*_1_, the line joining the normal and anchor node can have two possible coordinates *P*_1_ and *P*′_1_. Similarly, from the sensing range of *B*_2_, another two possible coordinates *P*_2_ and *P*′_2_ can be obtained. Then, the normal node compares the distance between each combination of points, *i.e.*, *P*_1_ with *P*_2_ or *P*′_2_ with *P*′_1_ or any other pairs. Finally, it chooses the pair of points having minimum distance or very negligible distance.

As shown in Figure 3(a), obviously points *P*′_1_ and *P*′_2_ are selected as the most possible location of the nodes. As shown in Figure 3(b), if more than two beacon packets are received from three different beacon nodes, normal node continues to update the coordinate table and use the same procedure to compute the possible coordinates *P*_3_ and *P*′_3_ and determines the correct coordinate. If more than three beacon packets are received from different beacon nodes after the waiting time is elapsed, a normal node selects three of all entity in the table having smallest RSSI value, as the error measurement of distance increases if the RSSI value is increased. By choosing the smallest RSSI value, the percentage of localization error can be minimized. In case, one beacon packet is received by the normal node during its waiting time, it implies that there is only one entity in the table with two possible coordinates *P*_1_ and *P*′_1_. In this case, a normal node randomly chooses one of the two possible coordinates as its estimated location.

The detail procedure of executing the localization algorithm is given in Table 2. Since there may be slight differences between the final coordinates, the error estimation and correction methods as described in Subsection 3.3 can be used to find the most accurate location of a normal node.

### 3.3. Localization Error Determination

It is to be noted that we propose the distributed localization algorithm taking three different types of nodes. We use location information of at least one or at most three beacon nodes to calculate the location of normal nodes. The anchor nodes do not provide location information neither to beacon nor to normal nodes. In our algorithm, they can provide only angle information to the normal nodes. Since we consider at most three beacon nodes to calculate location of the normal nodes, it could be possible that a normal node may calculate three different locations from the RSSI values received from three different beacon nodes. Hence, we use here the probability distribution function (PDF) to determine the probability of wrong identification of a transmission from the beacon node *A* as if it is originating either from the location of beacon node *B* or *C*. Besides, we also give the analytical methods to determine the probability of wrong identification from the beacon nodes *B* and *C* with respect to the beacon node *A*.

**Figure 4.**
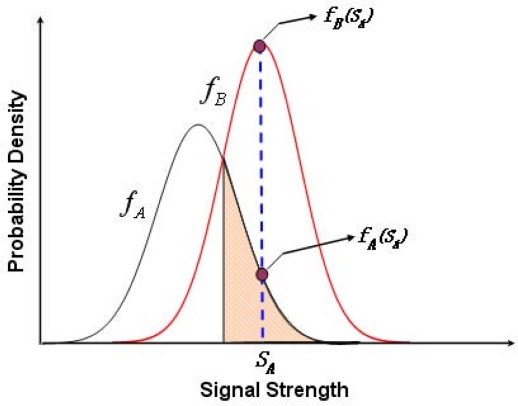
Probability density functions of RSSI received from beacon node *A*.

It is obvious that only one pair of coordinate is used as the location of the normal node, if only one beacon node is used to calculate the location of the normal node. However, the presence of more beacon nodes can enhance the accuracy of the localization. Hence, we propose our system with two or three beacon nodes for error analysis as follows. Consider three beacon nodes *A*, *B* and *C* located at different positions but within the communication range of a normal node. Let *S_A_*, *S_B_* and *S_C_* be the received signal strength (RSSI) by a normal node from those beacon nodes *A*, *B* and *C*, respectively. *f_A_*, *f_B_* and *f_C_* are the probability density functions (PDF) of the received signal strength *S_A_*, *S_B_* and *S_C_*, respectively, which are received by a normal node. Taking *S_A_* as the RSSI value received by the normal node from beacon node *A* and using this value in *f_A_* and *f_B_*, we can calculate *f_A_*(*S_A_*) and *f_B_*(*S_A_*), respectively. In this process, if *f_A_*(*S_A_*) *< f_B_*(*S_A_*), the normal node wrongly decides that the transmission has originated from *B* instead of *A*. As shown in Figure 4, as an example, *f_B_*(*S_A_*) is determined from the RSSI value received from node *A* and the shaded area represents the probability of wrong identification, which can be expressed analytically as follows,

(8) $$P_{A\to B}=P\left(f_{A}\left(S_{A}\right)<f_{B}\left(S_{A}\right)\right)$$

and

(9) $$P_{A\to C}=P\left(f_{A}\left(S_{A}\right)<f_{C}\left(S_{A}\right)\right)$$

where *P_A→B_* is the probability of wrongly identifying a transmission arriving from beacon node *A* as if it originates from beacon node *B*. Similarly, *P_A→C_* is the probability of wrongly identifying a transmission arriving from beacon node *A* as if it originates from beacon node *C*.

It is to be noted that a normal node also receives RSSI value from other beacon nodes *B* and *C*. Taking *S_B_* as the RSSI value of beacon node *B*, the probability of wrong identification of a transmission arriving from beacon node *B* with respect to beacon nodes *A* and *C* can be expressed analytically as follows,

(10) $$P_{B\to A}=P\left(f_{B}\left(S_{B}\right)<f_{A}\left(S_{B}\right)\right)$$

and

(11) $$P_{B\to C}=P\left(f_{B}\left(S_{B}\right)<f_{C}\left(S_{B}\right)\right)$$

Similarly, the probability of wrong identification of a transmission arriving from beacon node *C* with respect to beacon nodes *A* and *B* can be expressed analytically as follows,

(12) $$P_{C\to A}=P\left(f_{C}\left(S_{C}\right)<f_{A}\left(S_{C}\right)\right)$$

and

(13) $$P_{C\to B}=P\left(f_{C}\left(S_{C}\right)<f_{B}\left(S_{C}\right)\right)$$

As given in Equations (8)–(13), probability of erroneous localization can be measured in a probabilistic location determination system, which can be used further to reduce the localization error as described in the following subsection.

### 3.4. Localization Error Reduction

**Figure 5.**
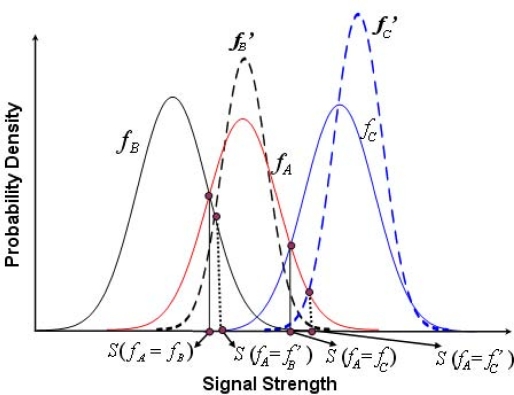
Probability density functions of signal strength received from three beacon nodes.

In this section, probabilistic methods for improving the localization accuracy of the normal nodes with respect to the locations of at most three beacon nodes are designed. Let *f_A_*, *f_B_* and *f_C_* be the probability density functions of the RSSI *S_A_*, *S_B_* and *S_C_*, received from the beacon nodes *A*, *B* and *C*, respectively. Suppose *μ_A_*, *μ_B_* and *μ_C_* are the mean and *σ_A_*, *σ_B_* and *σ_C_* are the standard deviations of *f_A_*, *f_B_* and *f_C_*, respectively. Let *μ_A_* *< μ_B_* and *μ_A_* *< μ_C_*. Let us define *S*(*f_A_* = *f_B_*) be the RSSI value when *f_A_*(*S*) = *f_B_*(*S*) and *S*(*f_A_* = *f_C_*) be the RSSI value when *f_A_*(*S*) = *f_C_*(*S*). As shown in Figure 5, for a given range of *S_A_*, *f_A_*(*S_A_*) *< f_B_*(*S_A_*) and *f_A_*(*S_A_*) *< f_C_*(*S_A_*), which implies that *S*(*f_A_* = *f_B_*) *< S_A_* *<* ∞ and *S*(*f_A_* = *f_C_*) *< S_A_* *<* ∞. Hence, the probability of getting an RSSI value in this range at the normal node from beacon node *B* is given by the following equation.

(14) $$P_{A\to B}=P\left(f_{A}\left(S_{A}\right)<f_{B}\left(S_{A}\right)\right)=\int_{S\left(f_{A}=f_{B}\right)}^{\infty }\;f_{A}\left(S\right)\cdot dS$$

Similarly, probability of getting an RSSI value in this range at the normal node from beacon node *C* is given by the following equation.

(15) $$P_{A\to C}=P\left(f_{A}\left(S_{A}\right)<f_{C}\left(S_{A}\right)\right)=\int_{S\left(f_{A}=f_{C}\right)}^{\infty }\;f_{A}\left(S\right)\cdot \mathrm{dS}$$

Here, *P_A_*_→_*_B_* represents the probability of identification of a beacon located at *A* as if it is the beacon *B* and *P_A_*_→_*_C_* represents the probability of identification of a beacon located at *A* as if it is the beacon *C. S*(*f_A_* = *f_B_*) represents the RSSI value at the normal node, where the PDFs from beacons *A* and *B* are equal to each other and *S*(*f_A_* = *f_C_*) represents the RSSI value at the normal node, where the PDFs from beacons *A* and *C* are equal to each other.

By considering a suitable method proposed in [22], the variance of the signal strength distribution at the normal node from the beacon node *B* is reduced to *σ*′*_B_*, where *σ*′*_B_* *< σ_B_*. Here, we define a new RSSI value for which *S*(*f_A_* = *f′_B_*) such that *S*(*f_A_* = *f_B_*) *< S*(*f_A_* = *f′_B_*). Hence, the probability of identification of location of beacon *A* as location *B* based on the new signal strength distribution from a transmitter located at *B* with reduced variance can be expressed in the following equation.

(16) $$P_{A\to B}^{\prime }=\int_{S\left(f_{A}=f_{B}^{\prime }\right)}^{\infty }\;f_{A}\left(S\right)\cdot \mathrm{dS}$$

Similarly, by reducing the variance of the signal strength distribution to *σ*′*_C_* at the normal node from beacon *C*, where *σ*′*_C_* *< σ_C_*, a new RSSI value could be defined so that *S*(*f_A_* = *f′_C_*), where *S*(*f_A_* = *f_C_*) *< S*(*f_A_* = *f′_C_*). Then the probability of identifying location of beacon *A* as location *C* based on the new signal strength distribution from a transmitter located at *C* with reduced variance can be expressed in the following equation.

(17) $$P_{A\to C}^{\prime }=\int_{S\left(f_{A}=f_{C}^{\prime }\right)}^{\infty }\;f_{A}\left(S\right)\cdot dS$$

Similarly, the probability of identification of location of beacon *B* as location *C* based on the new signal strength distribution from a transmitter located at *C* with reduced variance can be expressed in the following equation.

(18) $$P_{B\to C}^{\prime }=\int_{S\left(f_{B}=f_{C}^{\prime }\right)}^{\infty }\;f_{B}\left(S\right)\cdot \mathrm{dS}$$

Thus, the probability of identification of location of beacon *B* as location *A* based on the new signal strength distribution from a transmitter located at *A* with reduced variance can be expressed in the following equation.

(19) $$P_{B\to A}^{\prime }=\int_{S\left(f_{B}=f_{A}^{\prime }\right)}^{\infty }\;f_{B}\left(S\right)\cdot dS$$

Using the method of induction, the probability of identification of location of beacon *C* as location *A* based on the new signal strength distribution from a transmitter located at *A* with reduced variance can be expressed in the following equation.

(20) $$P_{C\to A}^{\prime }=\int_{S\left(f_{C}=f_{A}^{\prime }\right)}^{\infty }\;f_{C}\left(S\right)\cdot dS$$

And, the probability of identification of location of beacon *C* as location *B* based on the new signal strength distribution from a transmitter located at *B* with reduced variance can be expressed as

(21) $$P_{C\to B}^{\prime }=\int_{S\left(f_{C}=f_{B}^{\prime }\right)}^{\infty }\;f_{C}\left(S\right)\cdot dS$$

**Figure 6.**
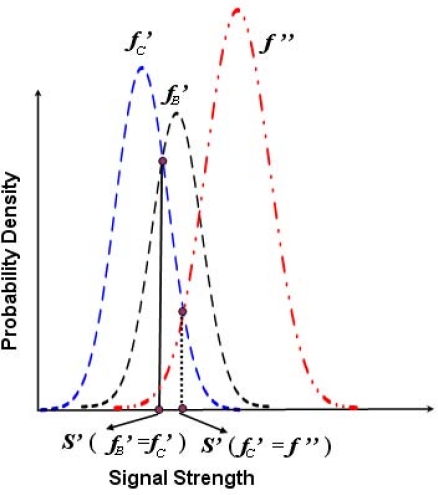
Probability density functions of signal strength received from three beacon nodes.

After getting *f′_B_* and *f′_C_*, as shown in Figure 6, and taking the reduced variance as discussed above, a new PDF *f*″ could be designed such that *S′*(*f′_B_* = *f′_C_*) *< S′*(*f′_C_* = *f*″). Similarly, after getting *f′_A_* and *f′_C_*, and taking the reduced variance, the new PDF *f*″ could be designed such that *S′*(*f′_A_* = *f′_C_*) *< S′*(*f′_C_* = *f*″). Finally, the probability of correctly identifying location of all beacons *A*, *B* and *C* at the normal node with reduced variance can be expressed in the following equation.

(22) $$P_{\mathrm{normal}}=\int_{S^{\prime }\left(f_{C}^{\prime }=f^{\prime \prime }\right)}^{\infty }\;f_{A}\left(S\right)\cdot \mathrm{dS}$$

where, *P_normal_* is the reduced probability of wrongly identifying a transmission from an object at location *A* as originating from location *C*. By considering three beacon nodes *A*, *B* and *C* at the same time, probability of error correction can be made as the combination among any two of those three beacon nodes, which can be similar to the above equation. Hence, by reducing in variance of each distributions, the location determination error could be reduced as discussed above.

## 4. Performance Evaluation

In this section, we evaluate the performance of our distributed localization algorithms. Detail description of the simulation setups and results are given as follows.

### 4.1. Simulation Setup

To analyze the performance of our algorithm, a rectangular monitoring region of size 200 × 300 *m*^2^ is taken and the algorithm is simulated using ns 2.33. The number of nodes deployed over the said monitoring region varies from 250 to 500 including normal, beacon and anchor nodes. In our simulation, 80% to 90% of the total number of deployed nodes is taken as the normal nodes without location information and rests are considered as the nodes with known location (beacon and anchor nodes). In our simulation, IEEE 802.15.4 MAC is considered as the medium access mechanism. Communication range of all normal nodes is fixed at 40 m and value of path loss exponent is set to 2. Each beacon node transmits beacon packet in an interval of 2 ms and the initial energy resource of each sensor node is considered as 50 joules, which is decreased by 0.3 joules in each transmission. Besides, Shadowing Model is used in our simulation to simulate the shadow effect of obstructions between the transmitter and receiver. In the simulation, the shadowing deviation of 10 dB and path loss (*β*) is taken 3, which are suitable for the shadowed outdoor environment [23,24].

### 4.2. Simulation Results

**Figure 7.**
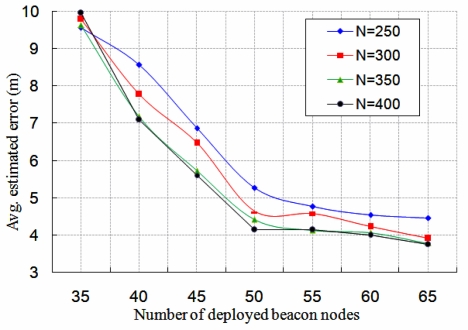
Average estimated localization error for different number of beacon nodes.

We first find out the average estimated localization error, which is defined as the square root of the mean-square error (RMSE) that is due to difference between the estimated coordinate and the real coordinate of a normal node. As shown in Figure 7, the average estimated localization error for different number of beacon nodes with fixed number of total nodes (*N*) is analyzed. In this simulation, the number of anchor nodes among the total number of nodes *N* is also fixed. From this figure, it is observed that the average estimated localization error decreases if number of beacon nodes increases. Besides, if more number of nodes *N* are deployed over the monitoring region, the average estimated localization error is also decreased. It is to be noted that the average localization error is more than 9 m when the number of beacon nodes is 35. Hence, in order to get more localization accuracy of the normal nodes, deployment of more beacon nodes is essential.

**Figure 8.**
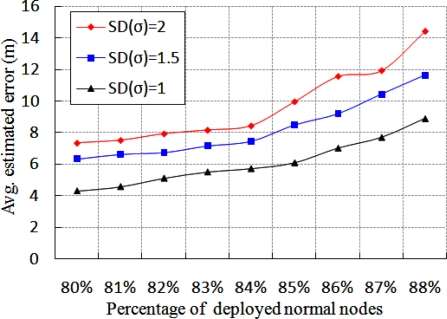
Effect of Standard Deviation (SD) on localization error for different number of normal nodes.

As discussed in Section 3, we use the path loss shadowing model as our propagation model given in Equation (3). In this model, *X_σ_* is a random variable with standard deviation *σ*, which affects the RSSI value and thereby influences the estimated average localization error. As shown in Figure 8, we simulated the percentage of deployed normal nodes with different standard deviation (*σ*) to study the average estimated localization error. The number of anchor and beacon nodes in this experiment is fixed. It is noticed that the average estimated localization error is more for large value of the standard deviation. It is quite reasonable, as the large value of standard deviation means the degree of probability distribution is large, and therefore the average localization error is increased. Besides, the estimated localization error increases if percentage of deployed normal nodes is increased. It is to be noted that the average localization error is increased in Figure 8 due to the effect of standard deviation (*σ*), only when 80%*∼*88% of normal nodes out of the total number of deployed nodes is considered in the simulation.

**Figure 9.**
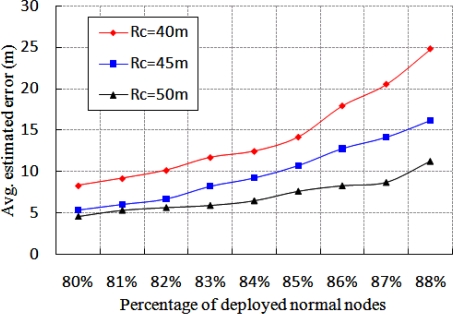
Average estimated localization error for different communication ranges.

Figure 9 indicates how the average estimated localization error is affected for different percentage of normal nodes. This experiment is carried out for different communication range of the beacon nodes with fixed number of anchor nodes, which is equal to 9. From this figure, it is found that the average estimated localization error is reduced, if communication range of the beacon nodes is increased. This situation happens, since most of the normal nodes can receive enough beacon packets to calculate their location and thereby reducing the localization error. However, if percentage of normal node increases, average estimated localization error also increases, which is compatible with the results given in Figure 8.

**Figure 10.**
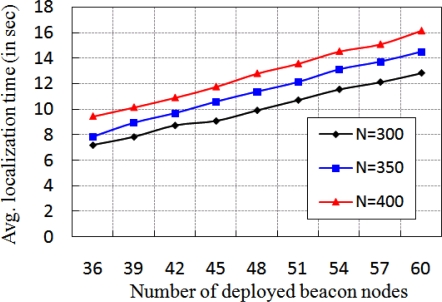
Average localization time for different number of beacon nodes.

**Figure 11.**
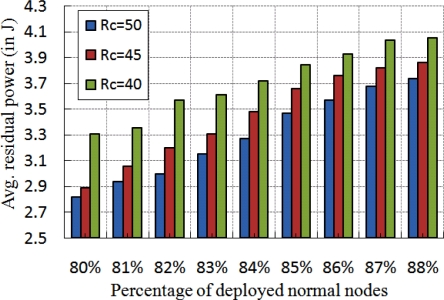
Average residual power for different number of beacon nodes.

In Figure 10, the localization time for different number of network size *N* with different number of beacon nodes is shown. Here, the localization time increases with increase in number of beacon nodes. This situation arises as a normal node waits for *T_n_* units and the communication time is also increased if number of beacon nodes increases. From Figure 10, it is interesting to note that the variation in localization time is much less although the node density changes. The analysis of average residual power for different communication range with different percentage of normal nodes is presented in Figure 11. In this experiment, first we measure the residual power of the beacon, anchor and normal nodes and then take the average of the total residual power. As shown in Figure 11, we observe that the average energy consumption is increased if number of normal nodes is increased. Besides, the residual power decreases if communication range of the normal nodes increases. This is because of the more power consumption due to higher communication range.

**Figure 12.**
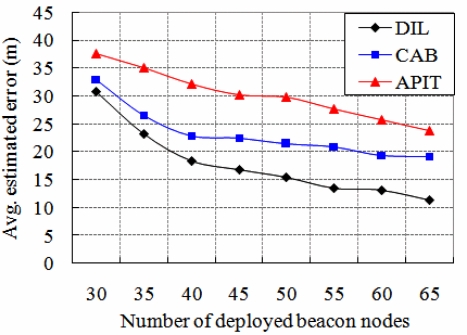
Comparison of average estimated localization error for different number of beacon nodes.

The comparison of the performance of our DIstributed Localization (DIL) algorithm is made with other similar localization algorithms CAB [5] and APIT [11]. The concentric anchor beacon (CAB) localization algorithm uses a small number of anchor nodes. Each anchor emits several beacon signals at different power levels, which are received by the sensors to calculate their location. We have considered CAB to compare with our simulation results, as we use three beacons that transmit signals to the normal nodes. The normal nodes calculate their location based on the RSSI values of the beacons. In APIT (Approximate Point In Triangle), a reference node sends location information periodically to the sensors. Then each sensor makes triangles based on the received location information of the reference nodes in order to calculate its own location. We compare APIT with our simulation results, as in our protocol, the beacon node transmits the location information to each normal node, which ultimately calculates its location. As shown in Figure 12, the average localization error of our algorithm is compared with CAB and APIT for different number of beacon nodes. It is observed that the average localization error decreases if number of beacon nodes increases. If the number of beacon nodes is about 40, the average estimated localization error of our scheme can have up to 18.36 % error, whereas CAB and APIT can have larger localization error, which is up to 22.82 % and 32.17 %, respectively. Obviously, DIL can have better performance over CAB and APIT. Besides, CAB can have limited improvement in localization error if number of beacon nodes is increased, but we can get better localization accuracy of normal nodes if number of beacons are increased in our protocol.

**Figure 13.**
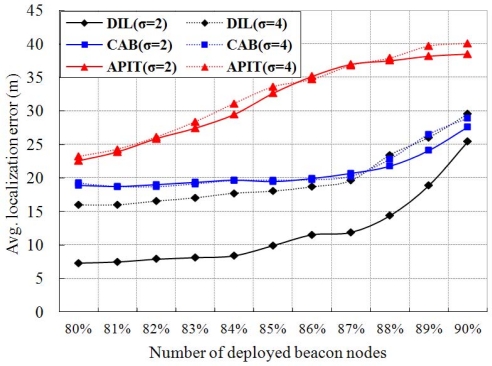
Comparison of average estimated localization error for different standard deviations.

**Figure 14.**
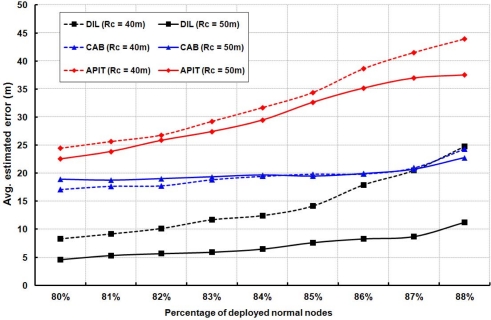
Comparison of average estimated localization error for different number of beacon nodes.

As shown in Figure 13, we simulate and compare with CAB and APIT to study the effect of different standard deviation (*σ*) on estimated localization error. It is noted that the standard deviation has slight effect on other two schemes, but have larger effect on our algorithm. It is reasonable in case of CAB and APIT as they do not use RSSI to find location of a node. Figure 14 shows the average estimated localization error with variation in communication range. Our algorithm as well as APIT scheme have better performance when the communication range increases. Since the communication range increases, it implies that more normal node can receive more beacon packets from different beacon nodes. Thus, the accuracy of localization is improved. However, CAB is not affected as the beacon nodes in CAB always transmit data with fixed power level. Beside, it is found that the average estimated localization error is decreased if the number of beacon nodes is increased.

**Figure 15.**
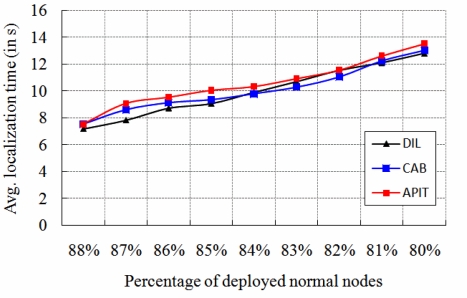
Comparison of localization time.

Figure 15 indicates the comparisons of localization time for different protocols, while different localization schemes are executed. The results show that DIL algorithm gives better performance on localization time, which is due to the limited transmission overheads. It is to be noted that, in our protocol, each anchor node and beacon nodes transmit packets to their neighbors only once.

## 5. Conclusions

In this paper, a novel distributed localization algorithm is proposed to find location of the normal nodes using only two or three beacon nodes. The localization error determination and error correction methods are proposed to give theoretical basis to the proposed algorithms. The advantage of our algorithm is that it can work even if only one beacon node provides location information to a normal node. From the performance evaluation of our algorithm, it is observed that our algorithms outperform over similar protocols. Besides, using the proposed method, location of the nodes can be calculated with the simplest ways with less time complexity, which is quite suitable for the memory and energy constraint sensors.

## Figures and Tables

**Table 1.** Coordinate table.

BN-ID	BN-loc.	RSSI	P-loc
*B*_1_	(*X*_*B*1_,*Y*_*B*1_)	*RSSI*_*B*1_	*P*_1_
*B*_2_	(*X*_*B*2_,*Y*_*B*2_)	*RSSI_B_*_2_	*P*′_2_
*B*_3_	(*X*_*B*3_,*Y*_*B*3_)	*RSSI*_*B*3_	*P*′_3_
*B*_4_	(*X*_*B*4_,*Y*_*B*4_)	*RSSI*_*B*4_	*P*_4_
...	...	...	...

**Table 2.** Distributed Localization (DIL) Algorithm.

**Initialize:**
Waiting time *T_n_* for each normal node;
All fields of coordinate table ={ϕ};
Start: Node deployment strategy;
**Do**
{
**For each Anchor node:**
Check: Neighbors of each normal nodes;
Measure: Angle information for each neighbor of normal nodes;
Transmit: Angle information to each normal nodes;
**For each Beacon node:**
Broadcast the beacon packets;
**For each Normal node:**
Setup: Waiting time *T_n_*;
**Do**
{
**Listen** to the network;
**If** (any beacon packet is received)
**Translate:** RSSI into Distance;
**Start location computation;**
Update the coordinate table;
**End If**
} **While** (*T_n_* is not expired);
Calculate: Final result from all entries of the table;
Output: Normal node’s location;
}
**End**
